# Preoperative remote consultation for a green bariatric surgery: can telemedicine be safe for patients and sustainable for the environment?

**DOI:** 10.3389/fsurg.2025.1604486

**Published:** 2025-07-07

**Authors:** Luigi Eduardo Conte, Michela Orsi, Alessandro Peruzzi, Chiara Fantozzi, Michela Campanelli, Domenico Benavoli, Bruno Sensi, Paolo Gentileschi

**Affiliations:** ^1^ Department of Surgical Science, University of Rome Tor Vergata, Rome, Italy; ^2^ Technical Department, Province of Viterbo, Viterbo, Italy; ^3^ Department of Bariatric and Metabolic Surgery, Maria Cecilia Hospital, GVM Care & Research, Cotignola, Ravenna, Italy

**Keywords:** green surgery, bariatric surgery, surgery, telemedicine, environment, obesity

## Abstract

**Background:**

The increasing emphasis on sustainability and digitalization has brought telemedicine to the forefront, particularly in bariatric surgery. This study evaluates the safety, clinical effectiveness and environmental impact of telemedicine for patients with obesity eligible for bariatric surgery.

**Materials and methods:**

A total of 338 patients underwent remote consultations via Zoom in 2023. Median age was 38 years (±4); 70% of patients were female. Mean BMI before surgery was 43 kg/m^2^ (±5). Surgical procedures included One Anastomosis Gastric Bypass (50%), Sleeve Gastrectomy (20%), Roux-en-Y Gastric Bypass (20%), revisional surgery (8%), and other procedures (2%). CO_2_ emissions were estimated based on the avoided travel, considering patient-reported transportation methods and distances. All patients were later evaluated in person before hospital admission. The primary outcome was environmental impact; the secondary outcome was whether remote evaluations influenced surgical indication or perioperative management.

**Results:**

Remote consultations reduced total travel emissions from 50,766.01 kg to 17.73 kg CO_2_, a 99.97% reduction (from 150.20 kg to 0.05 kg per patient). No patients required additional telemedicine or outpatient visits. Surgical indications and preoperative plans remained unchanged after in-person consultations. Postoperative complications occurred in 7.4% of patients, all minor and managed conservatively. There was no mortality. Median length of stay was 2.6 ± 3 days. At one-year follow-up, the mean BMI decreased to 29 kg/m^2^ (±3). In the revisional surgery group, percent excess weight loss reached 75.4% at one year.

**Conclusion:**

Telemedicine in bariatric surgery is safe, effective, and significantly reduces environmental impact. Remote consultations did not alter clinical decisions or outcomes. Wider adoption could benefit from dedicated digital tools to enhance patient care and sustainability.

## Introduction

As the years go by, sustainability and digitalization increasingly dominate public opinion and beyond (1, 2). Over time, people have become more aware that there is no “Planet B,” and thus, sustainability must be applied, as much as possible, to all fields, including medicine (3). COVID-19 was a terrible misfortune that forced the entire world to face unprecedented conditions, affecting everyone, especially the most vulnerable populations (4–6). Overnight, the entire global population had to deal with a pandemic and lockdowns (7–9). This terrible event did not only bring negative outcomes but also brought many new insights that we didn't have before. Examples include smart working (10, 11), digitalization and, above all, telemedicine (12–15). In times of extreme necessity, it has been shown that online medical consultations can be conducted remotely and safely. Moreover, considering the recent advent of artificial intelligence (16), the potential developments of this practice are truly endless. Returning to medicine, it must be said that obesity (17) is a serious and complex condition that cannot always be addressed with diet alone (18, 19). Obesity has been increasing at an alarming rate and, in recent years, has evolved into a global pandemic (20). In Italy, there has been an incredible upward trend in bariatric surgery procedures (21, 22). This is due both to the increasing number of bariatric patients and to the fact that bariatric surgery is becoming increasingly safe and is offering a wide diversity of options (23–26), allowing for patient-tailored procedures that meet the specific needs of this heterogeneous population. Telemedicine has attracted significant attention for its potential to enhance healthcare access while also reducing environmental impacts, with numerous studies backing these claims (27–31). This study aims to evaluate the application of telemedicine in patients with obesity initiating a bariatric surgery path, focusing on its clinical safety, efficiency, and environmental sustainability. Specifically, it investigates whether remote preoperative consultations can represent a valid alternative to in-person visits, ensuring comparable clinical outcomes while significantly reducing the carbon footprint associated with patient travel.

## Materials and methods

The study analyzes and considers the online medical consultations (initial consultation) conducted by a single experienced bariatric surgeon in 2023 (from 1/01/2023 and 31/12/2023). The study was conducted according to the international ethical recommendations on clinical research established by the Helsinki Declaration and in accordance with STROBE criteria (32).

### Study design

Retrospective single-centre study analyzing the environmental impact of video consultation (VC) in bariatric surgery.

All patients were referred to us for a VC, only after an initial evaluation by their primary care physician or a surgeon without specific expertise in bariatric surgery.

The consultations were conducted via video conference on Zoom and had an average duration of 20 min, comparable to in-person consultation. Online consultations are an alternative offered by our unit in place of in-person consultations conducted at Maria Cecilia Hospital – GVM Group located in Cotignola (RA). During the VC, we collected the patient's medical history, reviewed any available instrumental and laboratory test results, and, most importantly, assessed the patient's eligibility for bariatric surgery by considering a potential surgical approach. When investigations provided were not enough, new tests were asked and reviewed at the subsequent consultation. The necessary standard tests were: esophagogastroduodenoscopy, complete blood tests, psychiatric evaluation, spirometry in cases of obstructive sleep apnea syndrome, and cardiology consultation with echocardiography in the presence of cardiovascular issues.

This VC did not replace the initial in-person surgical consultation, which was conducted directly by an experienced bariatric surgeon during the pre-hospitalization phase (generally 2–5 days before the scheduled procedure). In this in-person visit, the eligibility of the patient with obesity for surgery was confirmed or rejected, and the appropriate type of surgical intervention was determined as well as the need for further investigations.

Patients were informed that any indications given on VC were not final but needed confirmation during in-person consultation.

Preoperative and postoperative data was also collected. Complications were classified according to Clavien-Dindo.

### Patients selection

All patients who had a remote consultation during the study period were included. There were no exclusion criteria. During the study period, patients were also seen in-person at our centre's outpatient clinic. The choice between remote vs. in-person consultation was based solely on patients' preference.

### Outcome measures

The primary outcome was the reduction of CO_2_ emissions following a telemedicine approach compared with a hypothetical in-person approach.

Secondary aim of the study is to assess whether there have been any changes in the clinical course of patients at the time of hospital admission following pre-hospital care. In particular, variables of study were confirmation of indication, need for adjunctive pre-operative exams and significant changes in planned management.

### Data analysis

To evaluate the environmental impact of each patient in terms of kg CO_2_ (kilograms of carbon dioxide) emitted, it was considered a round trip for all initial consultations, adding passenger cars and plane emissions, multiplied by the number of visits carried out in 2023 for each Italian region.

Microsoft Excel v. 16.90.2 was used for emissions calculation.

These following assumptions have been considered to collect data:

(a) Use of Google Maps on Google Chrome browser v. 128.0.6613.114 to calculate transport distances related to patients who reach Maria Cecilia Hospital.
(b) It was assumed that all patients living within 500 km from Maria Cecilia hospital traveled by car from the main city of the patient's region of origin, while above this cut-off it was assumed they would had traveled by plane to Bologna and then by car.
(c) Emission factor for cars is 161.92 gCO_2_/km, based on the EMEP/EEA air pollutant emission inventory guidebook 2019 and on the 2006 IPCC Guidelines relating to greenhouse gases (33). It is related to all passenger cars category in terms of engine power supply, selecting CO_2_ pollutant.
(d) Emission factor for plane is 123 gCO_2_/km for passenger (34).
(e) Emission factor for virtual meeting is 157.34 gCO_2_/h (35). The emission factor was related to an average duration of 20 min to in-person consultation for total emission calculation.

### Ethics

All patients provided informed consent for the treatment performed and for data collection. Prior to initiating the online video consultation, all patients accepted Zoom's privacy conditions. No images or videos of the consultation were recorded. According to local institutional review board, ethical approval for retrospective studies is not required.

## Results

The number of patients who were seen remotely during the study period was 338.

### Patients's demographics

Median age was 38 years (±4 years), 70% (n. 237) were females and 30% (n. 101) male. Mean BMI before surgery was 43 kg/m^2^ (±5 kg/m^2^) for the entire study population. 50% (n. 169) had underwent One Anastomosis Gastric Bypass, 20% (n. 68) Sleeve Gastrectomy, 20% (n. 68) Roux-en-Y Gastric Bypass, 8% (n. 27) revisional surgery (18 for bile reflux and 9 for weight regain) and 2% other types of bariatric surgery.

Baseline characteristics are summarized in Table 1.

**Table 1:** Patients's demographics.

Patient's characteristics	
Median age (±SD)	38 (±4) years
Sex (%)	F 237 (70%) – M 101 (30%)
BMI before surgery	43 kg/m^2^ (±5 kg/m^2^)
Type of bariatric surgery	
One anastomosis gastric bypass	169 (50%)
Sleeve gastrectomy	68 (20%)
Roux-en-Y gastric bypass	68 (20%)
Revisional surgery	27 (8%)
Other types of bariatric surgery	6 (2%)

### Primary outcome: emissions

Total travel emissions from the 338 patients in this study would have amounted to 50,766.01 kg CO_2_ (150.20 kg CO_2_ on average per patient). By conducting the outpatient visits remotely, their environmental impact was reduced to 17.73 kg CO_2_ (0.05 kg CO_2_ on average per patient). The percentage of reduction is thus −99.97% (Tables 2–4).

**Table 2:** Travel-related CO_2_ emissions by Italian region.

Region	Car emission factor [gCO_2_/km]	Plane emission factor [gCO_2_/p*km]	Visits/year	Distance to MCH (km)	Travel emissions (kgCO₂)
Sicilia	161.920	123.000	182	1,261	34,873.92
Calabria	161.920	123.000	12	949	2,411.56
Lombardia	161.920	123.000	7	269	609.78
Piemonte	161.920	123.000	6	385	748.05
Lazio	161.920	123.000	21	369	2,509.38
Toscana	161.920	123.000	16	133	689.11
Abruzzo	161.920	123.000	23	355	2,644.09
Puglia	161.920	123.000	14	628	2,217.67
Emilia-Romagna	161.920	123.000	21	44	299.22
Campania	161.920	123.000	10	564	1,301.15
Umbria	161.920	123.000	7	199	451.1
Veneto	161.920	123.000	9	202	588.73
Liguria	161.920	123.000	5	326	527.85
Sardegna	161.920	123.000	3	–	704.0
Friuli -Venezia Giulia	161.920	123.000	1	306	99.09
Molise	161.920	123.000	0	487	0.0
Marche	161.920	123.000	0	187	0.0
Trentino - Alto Adige	161.920	123.000	1	282	91.32
Basilicata	161.920	123.000	0	624	0.0
Total					**50,766**.**01**

**Table 3:** Videoconferencing-related CO_2_ emissions by Italian region.

Region	Visits/year	First visit duration (h)	VC emission factor [gCO2/h]	VC emissions (kgCO₂)
Sicilia	182	0.33	157,34	9.55
Calabria	12	0.33	157,34	0.63
Lombardia	7	0.33	157,34	0.37
Piemonte	6	0.33	157,34	0.31
Lazio	21	0.33	157,34	1.1
Toscana	16	0.33	157,34	0.84
Abruzzo	23	0.33	157,34	1.21
Puglia	14	0.33	157,34	0.73
Emilia-Romagna	21	0.33	157,34	1.1
Campania	10	0.33	157,34	0.52
Umbria	7	0.33	157,34	0.37
Veneto	9	0.33	157,34	0.47
Liguria	5	0.33	157,34	0.26
Sardegna	3	0.33	157,34	0.16
Friuli -Venezia Giulia	1	0.33	157,34	0.05
Molise	0	0.33	157,34	0.0
Marche	0	0.33	157,34	0.0
Trentino - Alto Adige	1	0.33	157,34	0.05
Basilicata	0	0.33	157,34	0.0
Total				**17**.**73**

**Table 4:** Comparison of travel vs videoconference emissions.

Region	Travel emissions (kgCO₂)	VC emissions (kgCO₂)
Sicilia	34,873.92	9.55
Calabria	2,411.56	0.63
Lombardia	609.78	0.37
Piemonte	748.05	0.31
Lazio	2,509.38	1.1
Toscana	689.11	0.84
Abruzzo	2,644.09	1.21
Puglia	2,217.67	0.73
Emilia-Romagna	299.22	1.1
Campania	1,301.15	0.52
Umbria	451.1	0.37
Veneto	588.73	0.47
Liguria	527.85	0.26
Sardegna	704.0	0.16
Friuli -Venezia Giulia	99.09	0.05
Molise	0.0	0.0
Marche	0.0	0.0
Trentino - Alto Adige	91.32	0.05
Basilicata	0.0	0.0
Total	**50,766**.**01**	**17**.**73**

### Secondary outcome: telemedicine and clinical decision

All patients were evaluated in person during pre-hospitalization. No patient required additional telemedicine or outpatient follow-up visits before surgery. The indication for surgery was never changed after in-person consultations nor was patient management in terms of need for additional investigations. The current status of the patient was never found to be significantly different to that estimated by remote evaluation.

### Postoperative outcomes

Postoperative outcomes are summarized in Table 5.

**Table 5:** Postoperative outcomes.

Complications and length of stay	
Median length of stay (±SD)	2.6 (±3) days
Mortality (%)	0 (0%)
Major complications	0 (0%)
Minor complications (CD 1 or 2)	25 (7.4%)
Succes of bariatric surgery	
BMI after surgery	29 kg/m^2^ (±3 kg/m^2^)
%EWL in the revisional group	43.7% at six months 75.4% at one year
Telemedicine	
Change of indication for surgery after in-person consultation	0 (0%)
Additional investigations required after in-person consultation	0 (0%)

#### Complications and length of stay

Mortality was nil and there were no major postoperative complications. Minor complications were 7.4% (n. 25) and included postoperative anemia (n. 16), melena (n. 4), wound infections (n. 3) and postoperative fistula (n. 2), all of which were treated conservatively. Median length of stay was 2.6 ± 3 days.

#### Success of bariatric surgery

All 338 patients correctly attended follow-up of minimun one year. The mean drop in BMI was significant, mean BMI after surgery was 29 kg/m^2^ (±3 kg/m^2^) for the entire study population. Revisional bariatric surgery was successful in 100% of cases. The mean of the percent excess weight loss in the revisional group was 43.7% at six months and 75.4% at one year.

## Discussion

Video Consultations are a hot topic in many countries. The advantages are clear and are further demonstrated in this study on bariatric surgery patients: a great impact on emissions and no impact on patient care. However, the latter point is of utmost importance and is strictly regulated in many countries. In Italy, VCs are limited by legislation (36–40): in particular, VC should not replace in-person physical examination, while it could be used as a “preliminary” consultation, only for elective cases. VCs in urgent settings or as a sole means of patient evaluation is thus illegal. While this may limit the utility of VC, as patients will need in-person visit regardless, we believe the existing laws are logical and common sense and devised for patient safeguard. To act in the safest possible way but to exploit the benefits of VC, we structured the process accordingly: our patients were all visited in person by a physician prior to VC (normally their general practicioner). Furthermore, the bariatric in-person consultation was performed on the day of pre-hospitalization. In this way, all patients were guaranteed maximum safety while taking advantage of environmental impact of VCs. The value of VCs in permitting high-standard patient care, was confirmed by our secondary outcome: in-person consultation never changed patient management. It may be correct to conclude that telemedicine in bariatric surgery, is a safe and effective primary consultation method which can be highly reliable in correctly directing patient management. The usefulness of this technique may be particularly important in patients living in remote areas and those with limited mobility, as it is often the case with bariatric patients. Regarding emissions, results are in line with other studies that report significant environmental impact reductions by using video conferences instead of face-to-face meeting (41). It is evident that conducting outpatient visits online would significantly reduce the environmental impact of medical practice. Conducting a telemedicine visit would eliminate all emissions directly associated with the patients' travel for this essential medical practice. Although it is not the only possible option, it should be thoroughly considered. Telemedicine offers the advantage of significantly reducing environmental impact, minimizing downtime between appointments, eliminating exhausting waits in crowded and often uncomfortable spaces, allowing for flawless collection of medical history, and offering the convenience of remote work for surgeons. Telemedicine has garnered significant attention for its potential to improve healthcare access while reducing environmental impacts, and numerous studies support these claims (26–31). Key environmental impacts, include:

1. Reduction in Carbon Emissions: Telemedicine significantly cuts down on transportation-related emissions. By eliminating the need for patients and healthcare providers to travel for appointments, telemedicine reduces the carbon footprint. For example, a study from the UK's NHS found that telemedicine consultations could save millions of patient miles annually, translating to large reductions in greenhouse gas emissions (30).
2. Decreased Energy Consumption: Hospitals and clinics are energy-intensive, with high demands for lighting, heating, air conditioning, and equipment. Shifting some of these consultations to a virtual platform reduces the need for large, continuously operational facilities. Several reports have noted energy savings due to lower facility usage.
3. Reduced Resource Usage: With fewer in-person visits, there's a reduced need for disposable medical supplies such as gloves or paper. Studies have shown that a single telemedicine visit can save a substantial amount of healthcare materials, further decreasing environmental burdens.
4. Waste Reduction: Physical healthcare facilities generate significant amounts of waste, including hazardous materials. By minimizing foot traffic through virtual consultations, telemedicine helps cut down on medical and non-medical waste production.
5. Potential Drawbacks: While the environmental benefits of telemedicine are clear, it is important to consider the digital infrastructure's impact. The use of servers, data centers, and electronic devices to support telemedicine platforms consumes energy and generates e-waste. However, studies indicate that the environmental cost of the digital infrastructure is still significantly lower than the emissions saved by reducing travel and in-person healthcare services.

Overall, telemedicine has strong support as an environmentally friendly alternative to traditional in-person healthcare, backed by numerous studies on its ability to reduce emissions, waste, and resource consumption, while expanding access to medical services. However, telemedicine cannot be seen as a cure-all solution. As is well known, every patient is different and requires personalized care based on their individual characteristics (42–48). One of the main issues today in Italy is the absence of a program, app, or website where patients can upload their lab or imaging results, allowing the surgeon to analyze them in-depth. Another issue is that it does not allow for a physical examination of the patient (49). Moreover, it should be considered that telemedicine can only be applied equitably if internet access is truly widespread. It cannot be assumed that all consultations can be conducted via telemedicine, as this would risk excluding the more vulnerable segments of the population who lack internet access. Additionally, elderly individuals, who may not be familiar with digital tools, could also be disadvantaged. In fact, while this consideration may be applicable in Italy, it becomes difficult to apply in countries where internet access is not as widespread. Additionally, there is a risk of losing the doctor-patient relationship, a cornerstone of this profession (50). These, however, are not insurmountable problems. The first issue could easily be solved by creating a program, app, or website specifically designed for these online visits, where patients could upload all their test results (both instrumental and otherwise) before the visit, allowing the surgeon to review them even before seeing the patient online. Pre-hospitalization, being mandatory, also allows the surgeon to conduct the required physical examination and strengthen the doctor-patient relationship that began online. Therefore, implementation of telemedicine should be encouraged and facilitated by technological development. It is foreseeable that artificial intelligence (24–28) could help us in creating an app, program, or website that, through a well-designed questionnaire, would allow the patient to quickly receive eligibility and recommendation for bariatric surgery. It is becoming increasingly evident that we are entering the era of artificial intelligence, which is steadily gaining traction and recognition, not only among patients, but even more so among healthcare professionals (51, 52). This evolving awareness is fostering a conceptual shift, whereby AI is no longer perceived as a competitor, but rather as a strategic ally in the advancement of patient care (53). However, this may remain a taboo/utopia for some time. Limitations of the study include its retrospective, single-center design, the non-comparative nature. While the assumptions concerning transport modalities may appear simplified, they were carefully considered during the study design phase in collaboration with statisticians and engineers. In the Italian context, structural limitations, such as insufficient railway coverage and a lack of widespread electric vehicle charging infrastructure, pose significant barriers to the use of more sustainable transportation options. This is particularly relevant in regions like Sicily, which accounted for the highest proportion of patients in this study and where connectivity challenges are especially pronounced. Consequently, calculations were based on the most commonly used modes of transport reported by patients, namely, private vehicles and, when feasible, air travel. Given the heterogeneous geographic distribution of the study population and the variability in transport accessibility, a standardized approach was necessary to ensure methodological consistency and minimize potential bias in estimating travel-related emissions. The analysis was based on standardized assumptions and secondary data from authoritative sources (e.g., IPCC guidelines). Travel distances were estimated via Google Maps and emission factors were treated as fixed values without probabilistic variation. Therefore, individual variability and uncertainty ranges were not incorporated. Additionally, the analysis focused only on travel-related emissions, excluding other environmental impacts. Future research should include patient-level data, broader indicators, and uncertainty analyses to enhance precision and applicability. Furthermore, future studies should aim to compare patient outcomes after VC and in-person consultations, with a particular emphasis on prospective and multicentric studies to strengthen and broaden current evidence.

In conclusion conducting bariatric surgery outpatient visits remotely significantly reduces the environmental impact of the hospital sector. The practice appears safe and reliable, provided an in-person consultation is also performed before surgery. Today, a consensus of experts is probably needed to define the possible applications and limitations of this practice. For telemedicine to be safely implemented, dedicated programs, apps, or websites are needed to address the challenges of this approach.

## Data Availability

The raw data supporting the conclusions of this article will be made available by the authors, without undue reservation.

## References

[1] Rising J Tedesco M Piontek F Stainforth DA. The missing risks of climate change. Nature. (2022) 610(7933):643–51. 10.1038/s41586-022-05243-6 36289386

[2] Carleton TA Hsiang SM. Social and economic impacts of climate. Science. (2016) 353(6304):aad9837. 10.1126/science.aad9837 27609899

[3] National Institute for Health and Care Research Global Health Research Unit on Global Surgery. Reducing the environmental impact of surgery on a global scale: systematic review and co-prioritization with healthcare workers in 132 countries. Br J Surg. (2023) 110(7):804–17. 10.1093/bjs/znad092 37079880 PMC10364528

[4] Rottoli M Bernante P Belvedere A Balsamo F Garelli S Giannella M How important is obesity as a risk factor for respiratory failure, intensive care admission and death in hospitalised COVID-19 patients? Results from a single Italian centre. Eur J Endocrinol. (2020) 183(4):389–97. 10.1530/EJE-20-0541 32674071 PMC9494325

[5] Rossi AP Muollo V Dalla Valle Z Urbani S Pellegrini M El Ghoch M The role of obesity, body composition, and nutrition in COVID-19 pandemia: a narrative review. Nutrients. (2022) 14(17):3493. 10.3390/nu14173493 36079751 PMC9458228

[6] Manzia TM Sensi B Conte LE Siragusa L Angelico R Frongillo F Evaluation of humoral response following SARS-CoV-2 mRNA-based vaccination in liver transplant recipients receiving tailored immunosuppressive therapy. J Clin Med. (2023) 12(21):6913. 10.3390/jcm12216913 37959382 PMC10650358

[7] Daly M Robinson E. Depression and anxiety during COVID-19. Lancet. (2022) 399(10324):518. 10.1016/S0140-6736(22)00187-8 35123689 PMC8813060

[8] COVID-19 Mental Disorders Collaborators. Global prevalence and burden of depressive and anxiety disorders in 204 countries and territories in 2020 due to the COVID-19 pandemic. Lancet. (2021) 398(10312):1700–12. 10.1016/S0140-6736(21)02143-7 34634250 PMC8500697

[9] Taquet M Holmes EA Harrison PJ. Depression and anxiety disorders during the COVID-19 pandemic: knowns and unknowns. Lancet. (2021) 398(10312):1665–6. 10.1016/S0140-6736(21)02221-2 34634251 PMC9754944

[10] Marino L Capone V. Smart working and well-being before and during the COVID-19 pandemic: a scoping review. Eur J Investig Health Psychol Educ. (2021) 11(4):1516–36. 10.3390/ejihpe11040108 34940386 PMC8700761

[11] Runfola M Fantola G Pintus S Iafrancesco M Moroni R. Telemedicine implementation on a bariatric outpatient clinic during COVID-19 pandemic in Italy: an unexpected hill-start. Obes Surg. (2020) 30(12):5145–9. 10.1007/s11695-020-05007-z 32981003 PMC7519889

[12] Katz C Robles N Novillo-Ortiz D Saigí-Rubió F. Selection of criteria for a telemedicine framework for designing, implementing, monitoring and evaluating telemedicine interventions: validation using a modified Delphi process. Digital Health. (2024) 10:20552076241251951. 10.1177/20552076241251951 38726219 PMC11080763

[13] Coldebella B Armfield NR Bambling M Hansen J Edirippulige S. The use of telemedicine for delivering healthcare to bariatric surgery patients: a literature review. J Telemed Telecare. (2018) 24(10):651–60. 10.1177/1357633X18795356 30343656

[14] Wang CD Rajaratnam T Stall B Hawa R Sockalingam S. Exploring the effects of telemedicine on bariatric surgery follow-up: a matched case control study. Obes Surg. (2019) 29(8):2704–6. 10.1007/s11695-019-03930-4 31134477

[15] Gunter RL Chouinard S Fernandes-Taylor S Wiseman JT Clarkson S Bennett K Current use of telemedicine for post-discharge surgical care: a systematic review. J Am Coll Surg. (2016) 222(5):915–27. 10.1016/j.jamcollsurg.2016.01.062 27016900 PMC5660861

[16] Bays HE Fitch A Cuda S Gonsahn-Bollie S Rickey E Hablutzel J Artificial intelligence and obesity management: an obesity medicine association (OMA) clinical practice statement (CPS) 2023. Obes Pillars. (2023) 6:100065. 10.1016/j.obpill.2023.100065 37990659 PMC10662105

[17] Campbell LA Kombathula R Jackson CD. Obesity in adults. JAMA. (2024) 332(7):600. 10.1001/jama.2024.5126 39102238

[18] Jenkins M Kurian M Moore R. Measuring outcomes in the treatment of obesity. JAMA Surg. (2024) 159(3):314. 10.1001/jamasurg.2023.6274 38055228

[19] Schoel LJ Telem DA. Gastric bypass vs diet-the need for contemporary comparisons. JAMA Surg. (2024) 159(9):980–1. 10.1001/jamasurg.2024.2114 38959018

[20] NCD Risk Factor Collaboration (NCD-RisC). Worldwide trends in underweight and obesity from 1990 to 2022: a pooled analysis of 3663 population-representative studies with 222 million children, adolescents, and adults. Lancet. (2024) 403(10431):1027–50. 10.1016/S0140-6736(23)02750-2 38432237 PMC7615769

[21] Gentileschi P Sensi B Siragusa L Sorge R Rispoli E Angrisani L Evolution of bariatric surgery in Italy in the last 11 years: data from the SICOB yearly national survey. Obes Surg. (2023) 33(3):930–7. 10.1007/s11695-022-06435-9 36690866 PMC9871429

[22] Boru CE Marinari GM Olmi S Gentileschi P Morino M Anselmino M Trends and safety of bariatric revisional surgery in Italy: multicenter, prospective, observational study. Surg Obes Relat Dis. (2023) 19(11):1270–80. 10.1016/j.soard.2023.05.009 37391349

[23] De Luca M Shikora S Eisenberg D Angrisani L Parmar C Alqahtani A Scientific evidence for the updated guidelines on indications for metabolic and bariatric surgery (IFSO/ASMBS). Obes Surg. (2024) 34(11):3963–4096. Advance online publication. 10.1007/s11695-024-07370-7 39320627 PMC11541402

[24] National GL. Available online at: https://www.sicob.org/00_materiali/Linee_Guida_SICOB_2023.pdf (Accessed January 04, 2025).

[25] De Luca M Zappa MA Zese M Bardi U Carbonelli MG Carrano FM Development of the Italian clinical practice guidelines on bariatric and metabolic surgery: design and methodological aspects. Nutrients. (2022) 15(1):189. 10.3390/nu15010189 36615848 PMC9823862

[26] De Luca M Silverii A Zese M Galasso G Bellini R Carbonelli MG Upcoming Italian clinical practice guidelines on endoscopic bariatric treatment of overweight and obesity: design and methodological aspects. Updates Surg. (2024) 76(5):1865–77. Advance online publication. 10.1007/s13304-024-01843-1 38985376

[27] Thiel CL Mehta N Sejo CS Qureshi L Moyer M Valentino V Telemedicine and the environment: life cycle environmental emissions from in-person and virtual clinic visits. NPJ digital Medicine. (2023) 6(1):87. 10.1038/s41746-023-00818-7 37160996 PMC10169113

[28] Qin RX Velin L Yates EF El Omrani O McLeod E Tudravu J Building sustainable and resilient surgical systems: a narrative review of opportunities to integrate climate change into national surgical planning in the western Pacific region. Lancet Reg Health West Pac. (2022) 22:100407. 10.1016/j.lanwpc.2022.100407 35243461 PMC8881731

[29] Holmner A Ebi KL Lazuardi L Nilsson M. Carbon footprint of telemedicine solutions–unexplored opportunity for reducing carbon emissions in the health sector. PLoS One. (2014) 9(9):e105040. 10.1371/journal.pone.0105040 25188322 PMC4154849

[30] Purohit A Smith J Hibble A. Does telemedicine reduce the carbon footprint of healthcare? A systematic review. Future Healthc J. (2021) 8(1):e85–91. 10.7861/fhj.2020-0080 33791483 PMC8004323

[31] Ravindrane R Patel J. The environmental impacts of telemedicine in place of face-to-face patient care: a systematic review. Future Healthc J. (2022) 9(1):28–33. 10.7861/fhj.2021-0148 35372776 PMC8966787

[32] Available online at: https://www.strobe-statement.org/ (Accessed January 04, 2025).

[33] Available online at: https://fetransp.isprambiente.it/#/ (Accessed January 04, 2025).

[34] IEA. Well-to-wheel (wake/wing) GHG intensity of motorised passenger transport modes, IEA, Paris (2022). Available online at: https://www.iea.org/data-and-statistics/charts/well-to-wheel-wake-wing-ghg-intensity-of-motorised-passenger-transport-modes-2, Licence: CC BY 4.0 (Accessed January 04, 2025).

[35] The overlooked environmental footprint of increasing Internet use Renee Obringer a,b, Benjamin Rachunok c,#, Debora Maia-Silva b,#, Maryam Arbabzadeh d,#, Roshanak Nateghi c,*, Kaveh Madani. 10.1016/j.resconrec.2020.105389

[36] Available online at: https://www.gazzettaufficiale.it/eli/id/2017/03/17/17G00041/sg (Accessed January 04, 2025).

[37] Available online at: https://www.statoregioni.it/it/conferenza-stato-regioni/sedute-2020/seduta-del-17122020/report/ (Accessed January 04, 2025).

[38] Available online at: https://www.gazzettaufficiale.it/eli/id/2022/06/22/22G00085/SG (Accessed January 04, 2025).

[39] Available online at: https://www.gazzettaufficiale.it/eli/id/2022/05/24/22A03098/sg (Accessed January 04, 2025).

[40] Available online at: https://www.gazzettaufficiale.it/eli/id/2022/11/02/22A06184/sg (Accessed January 04, 2025).

[41] Warland L Hilty L Küng J Reinhard J. Factsheet: Business Travel. 10.13140/RG.2.2.14496.58883. ] [D. Quack and M. Oley, “Environmental Advantages of Video Conferencing Systems - Results from a Simplified LCA,” in EnviroInfo 2002, Wien, 2002 (2016).

[42] Navarra G Komaei I Currò G Angrisani L Bellini R Cerbone MR Bariatric surgery and the COVID-19 pandemic: SICOB recommendations on how to perform surgery during the outbreak and when to resume the activities in phase 2 of lockdown. Updates Surg. (2020) 72(2):259–68. 10.1007/s13304-020-00821-7 32514743 PMC7278242

[43] Sileri P Franceschilli L Cadeddu F De Luca E D'Ugo S Tognoni V Prevalence of defaecatory disorders in morbidly obese patients before and after bariatric surgery. J Gastrointest Surg. (2012) 16(1):62–7. 10.1007/s11605-011-1705-5 21948149

[44] Gentileschi P Kini S Gagner M. Palliative laparoscopic hepatico- and gastrojejunostomy for advanced pancreatic cancer. Jsls. (2002) 6(4):331–8. 12500832 PMC3043447

[45] Guglielmi V D'Adamo M Menghini R Cardellini M Gentileschi P Federici M MicroRNA 21 is up-regulated in adipose tissue of obese diabetic subjects. Nutr Healthy Aging. (2017) 4(2):141–5. 10.3233/NHA-160020 28447068 PMC5389040

[46] Gagner M Gentileschi P. Hand-assisted laparoscopic pancreatic resection. Semin Laparosc Surg. (2001) 8(2):114–25. 10.1053/slas.2001.24185 11441400

[47] Tariciotti L D'Ugo S Manzia TM Tognoni V Sica G Gentileschi P Combined liver transplantation and sleeve gastrectomy for end-stage liver disease in a bariatric patient: first European case-report. Int J Surg Case Rep. (2016) 28:38–41. 10.1016/j.ijscr.2016.09.011 27677115 PMC5037123

[48] Musella M Vitiello A Susa A Greco F De Luca M Manno E Revisional surgery after one anastomosis/minigastric bypass: an Italian multi-institutional survey. Obes Surg. (2022) 32(2):256–65. 10.1007/s11695-021-05779-y 34973123 PMC8795019

[49] Hsiao V Chandereng T Huebner JA Kunstman DT Flood GE Tevaarwerk AJ Telemedicine use across medical specialties and diagnoses. Appl Clin Inform. (2023) 14(1):172–84. 10.1055/s-0043-1762595 36858112 PMC9977562

[50] Davenport A Brunn E Creswell M Sholklapper T Ringel N Gutman R. Exploring patient perspectives surrounding telemedicine versus in-person preoperative visits. Urogynecology. (2023) 29(6):545–51. 10.1097/SPV.0000000000001310 36701389

[51] Gaddi AV Lugaresi M. Telemedicine: a unique, univocal, and shared definition for everyone. Art Int Surg. (2024) 4:37–43. 10.20517/ais.2024.03

[52] Brochu BM Mirsky NA Thaller SR. Evaluating ChatGPT’s efficacy in addressing common patient questions in plastic surgery consultations. Art Int Surg. (2024) 4:411–26. 10.20517/ais.2024.61

[53] Bellini V Panizzi M Bignami EG. The health technology assessment in the artificial intelligence era: the AI surgical department. Art Int Surg. (2024) 4:44–7. 10.20517/ais.2024.10

