# Supplementary material for: Preoperative remote consultation for a green bariatric surgery: can telemedicine be safe for patients and sustainable for the environment?
Source: Front Surg. 2025 Jul 7;12:1604486. doi: 10.3389/fsurg.2025.1604486 (PMC12277319; doi:10.3389/fsurg.2025.1604486)
Supplement: Supplementary file 1 [file Datasheet1.pdf]

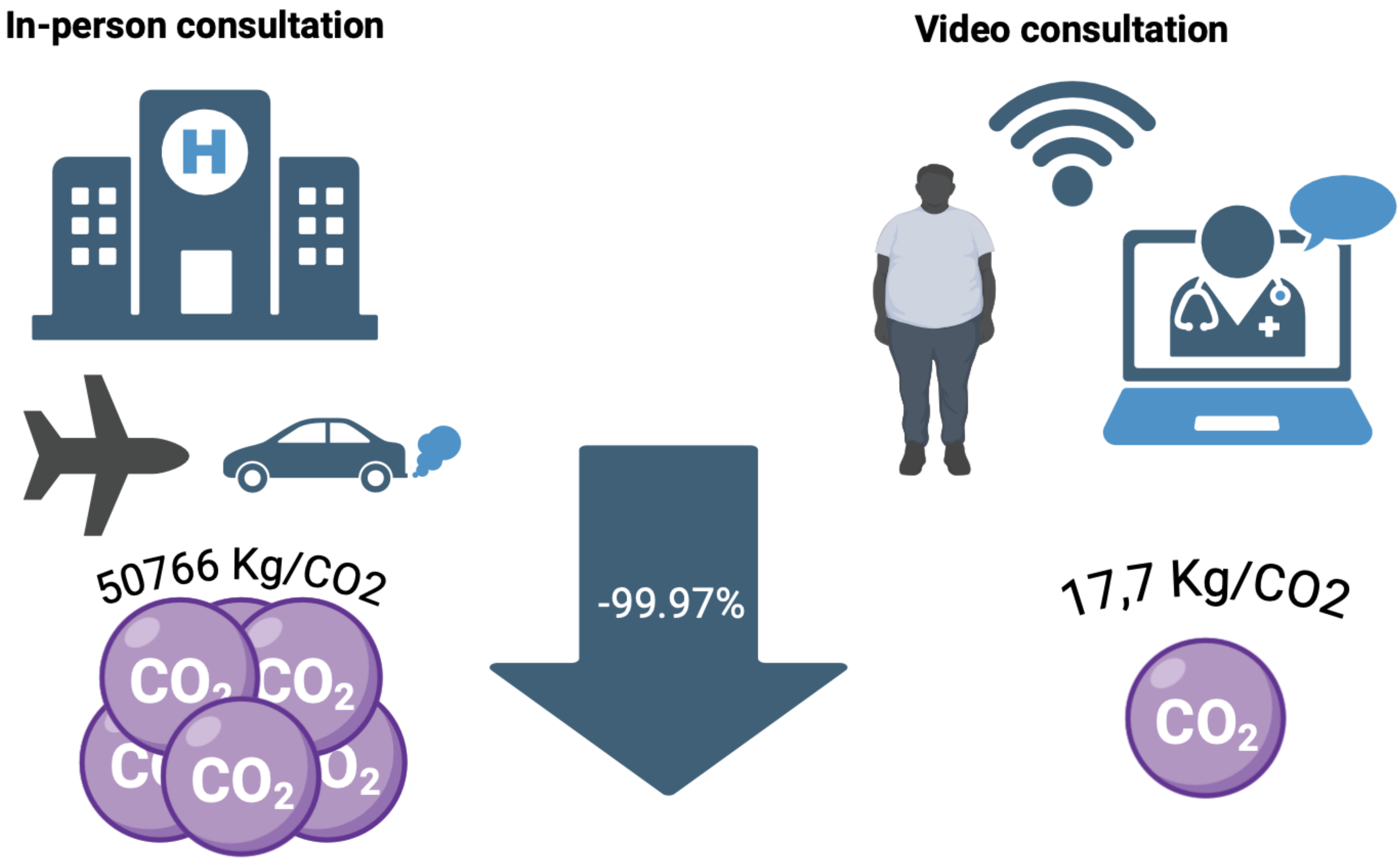

Remote consultations **reduced CO_2 emissions by 99.97%** with no impact on patients’ clinical outcomes or surgical decisions
